# Initial nitrous oxide, carbon dioxide, and methane costs of converting conservation reserve program grassland to row crops under no-till vs. conventional tillage

**DOI:** 10.1111/gcb.12216

**Published:** 2013-05-02

**Authors:** Leilei Ruan, G Philip Robertson

**Affiliations:** W.K. Kellogg Biological Station Department of Plant, Soil and Microbial Sciences Great Lakes Bioenergy Research Center, Michigan State UniversityHickory Corners, MI, 49060, USA

**Keywords:** carbon dioxide, conservation reserve program, global warming impact, greenhouse gas balance, methane, nitrous oxide, no-till, tillage

## Abstract

Around 4.4 million ha of land in USDA Conservation Reserve Program (CRP) contracts will expire between 2013 and 2018 and some will likely return to crop production. No-till (NT) management offers the potential to reduce the global warming costs of CO_2_, CH_4_, and N_2_O emissions during CRP conversion, but to date there have been no CRP conversion tillage comparisons. In 2009, we converted portions of three 9–21 ha CRP fields in Michigan to conventional tillage (CT) or NT soybean production and reserved a fourth field for reference. Both CO_2_ and N_2_O fluxes increased following herbicide application in all converted fields, but in the CT treatment substantial and immediate N_2_O and CO_2_ fluxes occurred after tillage. For the initial 201-day conversion period, average daily N_2_O fluxes (g N_2_O-N ha^−1^ d^−1^) were significantly different in the order: CT (47.5 ± 6.31, *n* = 6) ≫ NT (16.7 ± 2.45, *n* = 6) ≫ reference (2.51 ± 0.73, *n* = 4). Similarly, soil CO_2_ fluxes in CT were 1.2 times those in NT and 3.1 times those in the unconverted CRP reference field. All treatments were minor sinks for CH_4_ (−0.69 ± 0.42 to −1.86 ± 0.37 g CH_4_–C ha^−1^ d^−1^) with no significant differences among treatments. The positive global warming impact (GWI) of converted soybean fields under both CT (11.5 Mg CO_2_e ha^−1^) and NT (2.87 Mg CO_2_e ha^−1^) was in contrast to the negative GWI of the unconverted reference field (−3.5 Mg CO_2_e ha^−1^) with on-going greenhouse gas (GHG) mitigation. N_2_O contributed 39.3% and 55.0% of the GWI under CT and NT systems with the remainder contributed by CO_2_ (60.7% and 45.0%, respectively). Including foregone mitigation, we conclude that NT management can reduce GHG costs by ∼60% compared to CT during initial CRP conversion.

## Introduction

The USDA Conservation Reserve Program (CRP) builds contracts with agricultural landowners in the United States to retire highly erodible and environmentally sensitive cropland and pasture into perennial vegetation for periods ≥10 years. The program, established by the Food Security Act of 1985, is designed to reduce soil erosion, improve water and air quality, enhance wildlife populations, and to sequester carbon in soil and biomass. In 2007, as many as ∼15 million ha were enrolled, representing ∼9% of total US cropland (Economic Research Service (ERS), 2011; Farm Service Agency (FSA), 2012). Since then, enrolled land had decreased to ∼12 million ha in 2012, and an additional ∼4.4 million ha of land are in CRP contracts that will expire between 2013 and 2018 (Farm Service Agency (FSA), 2012). Higher prices for corn (*Zea mays* L.) and other crops and expanded biofuel production are expected to induce farmers to return CRP land to grain production (Du *et al*., 2008; Secchi *et al*., 2009). Many environmental benefits may subsequently be lost. Of particular concern are changes to greenhouse gas (GHG) emissions – fluxes of CO_2_, nitrous oxide (N_2_O) and methane (CH_4_) during and after conversion (CAST (Council for Agricultural Science & Technology), 2011).

Grassland conversion into crop production can accelerate both soil C and nitrogen (N) cycles, and results in significant GHG emissions. In particular, land conversion practices such as plowing can enhance soil organic matter oxidation, nitrification, and denitrification and substantially increase CO_2_ and N_2_O emissions (Pinto *et al*., 2004; Grandy & Robertson, 2006a; Nikièma *et al*., 2012). No-till (NT) offers the potential to attenuate such increases, but to date there have been no GHG comparisons of NT and conventional tillage (CT) during CRP conversion.

The effects of tillage on soil carbon are well known. Plowing mixes crop residues with the soil, increases the aeration of surface soil and reduces soil aggregation, all of which enhances organic matter decomposition and CO_2_ release (Haas *et al*., 1957; Buyanovsky & Wagner, 1998; Grandy & Robertson, 2006b; Regina & Alakukku, 2010). In contrast, the soil under NT is left undisturbed. More stable aggregates under NT protect soil organic carbon (SOC) from microbial decomposition and allow SOC storage (Six *et al*., 2000). Dolan *et al*. (2006), for example, reported that NT managed soil contained over 30% more SOC than CT soils to 20 cm after 23 years of NT. Syswerda *et al*. (2011) reported ∼11% higher SOC to 1 m depth under NT than CT after 12 years of NT. West & Post (2002) used a global database of 67 long-term agricultural experiments to estimate that conversion from CT to NT can annually sequester 48 ± 13 g C m^−2^ yr^−1^ in surface horizons. There is little evidence for statistically different changes at deeper depths (Kravchenko & Robertson, 2011). Following CRP conversion, Follett *et al*. (2009) reported no SOC change (0–30 cm depth) within 6.5 years after conversion of CRP grasslands to NT corn in Nebraska. Anken *et al*. (2004), however, reported that SOC (0–20 cm depth) decreased under both NT and CT similarly in Switzerland for the first 7 years after conversion of a 10 year old grassland to maize-winter wheat production.

Effects of CT on soil N_2_O emissions compared to NT are still in debate. Agricultural soil N_2_O emissions account for about 60% of global total anthropogenic N_2_O production (IPCC (Intergovernmental Panel on Climate Change), 2007) due to two microbial processes: denitrification and nitrification (Robertson & Groffman, 2007). Theoretically, NT can strongly affect both these processes through effects on soil water, carbon, pore space, and soil N concentrations. In practice, some studies have shown higher N_2_O emissions from NT than CT (e.g., Baggs *et al*., 2003; Rochette *et al*., 2008), with higher rates in NT mostly attributed to restricted soil aeration due to higher water content, which is conducive to denitrification. However, others have found lower emissions in NT than CT, attributed to improved soil structure and lower soil temperatures (e.g., Chatskikh & Olesen, 2007; Ussiri *et al*., 2009). Still others have found no difference between NT and CT (e.g., Robertson *et al*., 2000; Choudhary *et al*., 2002; Boeckx *et al*., 2011).

Methane oxidation is also affected by agricultural management. CH_4_ oxidation by methanotrophic bacteria in well-aerated soils is an important sink (5%, globally) for atmospheric CH_4_ IPCC (Intergovernmental Panel on Climate Change), 2007. In theory, a less disturbed soil structure and improved gas diffusion in NT should enhance the CH_4_ oxidation capacity of methanotrophic bacteria relative to CT (Six *et al*., 2004; Ussiri *et al*., 2009). However, studies to date have reported no significant NT effects on CH_4_ oxidation rates (Robertson *et al*., 2000; Jacinthe & Lal, 2005).

In an earlier study, Gelfand *et al*. (2011) reported that the conversion of CRP land to NT soybean production released significant amounts of CO_2_ and N_2_O and had little effect on CH_4_ oxidation rates. Here, we extend their results to examine the impact of CT practices on GHG fluxes during conversion. Specifically, we hypothesize that for the CRP conversion year, NT relative to CT will (i) attenuate N_2_O emissions; (ii) reduce C loss; and (iii) avoid the loss of CH_4_ oxidation. Furthermore, we evaluate the relative importance of each flux to the overall GHG cost of CRP conversion.

## Materials and methods

### Site description

Our experimental fields were located at the Great Lakes Bioenergy Research Center (GLBRC) scale-up field at the Marshall Farm of the Kellogg Biological Station (KBS) Long-term Ecological Research (LTER) site in southwest Michigan (42°26′N, 85°19′W, elevation 288 m). Annual precipitation is ∼890 mm with about half falling as snow, and the mean annual temperature is 9.7 °C. We conducted experiments in four separate fields enrolled in the CRP for 22 years beginning in 1987, when all fields were planted to the C3 grass smooth brome (*Bromus inermis* Leyss). Fields were 9–21 ha in size and within 1.8 km of one another. In 2009, three fields were converted to soybean (*Glycine max*) production. No fertilizers were applied although ammonium sulfate (0.33 kg N ha^−1^) was added to glyphosate as a surfactant. The fourth was reserved as a reference field unconverted.

Soils in all fields are mesic Typic Hapludalfs of three intermixed series: Boyer (loamy sand), Kalamazoo (fine-loamy) and Oshtemo (coarse-loamy) developed on glacial outwash. Prior to conversion, there were no significant differences among key soil properties including soil C and N contents, bulk density, and soil texture among the four CRP fields (Table 1) (http://data.sustainability.glbrc.org/).

**Table 1:** Soil physical and chemical properties (0–25 cm) of the four conservation reserve program (CRP) grassland fields prior to conversion. Means within columns marked with the same letters are not significantly different (*P* < 0.05)

	pH	Bulk density (g cm^−3^)	Nitrogen (g kg^−1^)	Carbon (g kg^−1^)	Sand (g kg^−1^ soil)	Silt (g kg^−1^ soil)	Soil texture
Field 1	6.4^a^	1.41^a^	2.10^a^	22.8^a^	664.0^a^	256.5^a^	Sandy loam
Field 2	6.7^a^	1.34^a^	1.81^a^	20.6^a^	697.0^a^	245.0^a^	Sandy loam
Field 3	6.3^a^	1.42^a^	1.72^a^	19.9^a^	688.1^a^	264.5^a^	Sandy loam
Reference	6.2^a^	1.41^a^	2.07^a^	22.5^a^	595.0^a^	328.5^a^	Sandy loam

### Experimental design and treatments

We established two replicated NT and CT plots in each of the three converted fields. We also randomly identified four replicate plots in the reference field. Treatment plots were 36 m by 9 m arranged in a randomized complete block design for a total of 16 plots (3 fields × 2 treatments × 2 replicate plots + 1 reference field × 4 replicate plots). Brome grass was killed at the converted fields on May 5, 2009, with glyphosate (N-phosphonomethyl, Syngenta, Greensboro, NC, USA) at a concentration of 2.85 kg ha^−1^ and killed grass residue was left in place. CT plots were tilled (25 cm deep) using a chisel plow and secondary tillage for leveling the surface on June 8. NT plots were left untilled, as was the remainder of each converted field. Soybeans (Pioneer 92M91) were planted on June 9 in all converted fields at a seeding rate of 355 680 seeds ha^−1^ using a no-till planter. The reference field was left undisturbed.

### Gas and soil measurement protocols

CO_2_, CH_4_, and N_2_O flux measurements were made using a static chamber approach as described by Hoben *et al*. (2011) between May 7 and November 24, 2009. We measured fluxes one to two times a week during the growing season to capture the temporal dynamics of gas fluxes influenced by different management activities, and then measured fluxes every 2 weeks after mid-September. Two chambers were installed in each treatment plot of the converted fields and one chamber was installed in each of four reference plots for a total of 36 chambers. Each chamber (28 cm diameter × 26 cm height) was equipped with a removable lid and septum. Chamber bases were embedded 5 cm into the soil for the duration of the study except during farm operations (tillage, soybean planting, and harvest), when chambers were removed from plots in the converted fields and replaced in the same spot within 2 h afterward.

For flux measurements, chamber lids were attached and headspace gas samples (10 ml) were collected four times with a 10 ml syringe from each chamber at intervals of approximately 15 min. Samples were stored over pressurized in 5.6 ml glass vials (Labco Ltd., High Wycombe, UK). Vials were returned to the laboratory where contents were analyzed using gas chromatography (Hewlett Packard 5890 Series II, Rolling Meadows, IL, USA) usually within 12 h of collection. Gases were separated on a Poropak Q column (1.8 m, 80/100 mesh) at 80 °C. CO_2_ was analyzed using an infrared gas absorption analyzer (LI-820 CO_2_ analyzer; LI-COR, Lincoln, NE, USA); CH_4_ was analyzed with a flame ionization detector at 300 °C; and N_2_O was analyzed with a ^63^Ni electron capture detector at 350 °C.

We also calculated the net ecosystem exchange (NEE) of CO_2_ at each field using data from Zenone *et al*. (2011) as reported on the KBS LTER website: http://lter.kbs.msu.edu/datatables/198. Four 3 m tall eddy covariance towers were located in the center of each field. The eddy covariance system included a LI-7500 open-path infrared gas analyzer (IRGA) (Li-Cor Biosciences, Lincoln, NE, USA), a CSAT3 three-dimensional sonic anemometer (Campbell Scientific Inc., Logan, UT, USA), and a CR5000 data logger (Campbell Scientific Inc.). The effective measurement radius of each eddy covariance tower was approximately 200 m and every 30 min NEE was calculated as the covariance of vertical wind speed and the concentration of CO_2_ as described in Zenone *et al*. (2011). Individual treatment plots (CT, NT, and reference) were outside the effective range of the towers such that NEE measurements were for NT soybeans (converted fields) or unconverted smooth brome grass (reference field). We calculated the NEE for CT soybean as NEE for NT soybean plus the difference we measured in soil CO_2_ fluxes between CT and NT treatments. This assumes that both CT and NT soybean treatments removed the same amount of CO_2_ from the atmosphere through photosynthesis as confirmed by similar yields for CT and NT treatments, and that CO_2_ fluxes from plant and herbivore respiration were similar for each treatment.

To estimate the global warming impact of conversion attributable to changes in CO_2_, CH_4_, and N_2_O fluxes, we multiplied fluxes of each gas by its global warming potential (GWP) to yield CO_2_ equivalents (Mg CO_2_e ha^−1^). For CO_2_, CH_4_, and N_2_O fluxes we used the IPCC 100-year horizon GWP factors of 1, 25, and 298, respectively (IPCC (Intergovernmental Panel on Climate Change), 2007).

At each gas sampling event, we measured soil temperature, gravimetric water content, ammonium (NH_4_^+^) and nitrate (NO_3_^−^) concentrations, BD, and water-filled pore space (WFPS%). Four 2.5 cm diameter cores (0–25 cm depth) were randomly collected within and between plant rows from each treatment plot. One core was then oven-dried to constant weight at 60 °C for 48 h to obtain gravimetric soil moisture (g water g^−1^ dry soil). The remaining three cores were composited and sieved to 4 mm. Three 10 g subsamples were then each extracted with 100 ml of 1 M KCl. Soil extracts were shaken by hand for 1 min, equilibrated overnight, reshaken and settled for 2 h before filtering through a 1 mm glass fiber syringe filter. Filtrates were stored in 7 ml polyethylene vials and frozen until analysis for NH_4_^+^ and NO_3_^−^ at a later date. Both analyses were performed on a Flow Solution IV continuous flow analyzer (OI Analytical, College Station, TX, USA) using colorimetric techniques.

Ion exchange resin strips were also used to estimate NH_4_^+^ and NO_3_^−^ availability (Qian & Schoenau, 1995). Two pairs of anion and cation strips (2.5 cm × 10 cm × 0.62 mm thick) (GE Power & Water, Trevose, PA, USA) were buried directly into the soil at each treatment plot. After 37 days, strips for each plot were collected and put into a 237 ml polyethylene cup. We added 35 ml of 2.0 M KCl per resin strip to each cup (i.e., 140 ml for four strips) and cups were then shaken at 40 rpm for 1 h on an orbital shaker (IKA KS 501, Wilmington, NC, USA). A 5 ml extract was stored in a 7 ml polyethylene vial and frozen until analysis for NH_4_^+^ and NO_3_^−^ as above.

Soil BD (0–25 cm depth) was measured on May 20, June 10, and November 20, 2009 using a fixed volume core (123 cm^3^) for each treatment plot. WFPS% was calculated as


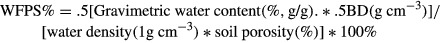


where soil porosity = 1–BD (g cm^−3^)/particle density (g cm^−3^). Particle density was assumed to be 2.65 g cm^−3^.

### Data analysis

Cumulative fluxes of gases were calculated by linear interpolation of daily fluxes between sample days in 2009. Data were analyzed using the PROC MIXED procedure in SAS 9.1 (SAS Institute, Cary, NC, USA). When comparing differences between CT and NT treatments, the experiment was analyzed as a randomized complete block design with the field as a blocking factor. Plots within the fields subjected to CT and NT treatments were used as experimental units for testing treatment effects. For comparisons between CT and the reference treatment or NT and the reference treatment the experimental unit was the field. To determine the relationship between daily fluxes (CO_2_, N_2_O, and CH_4_) and environmental factors such as soil temperature, gravimetric soil moisture, and soil total N, we performed multiple linear regressions (stepwise) using PROC REG and nonlinear regression using PROC NLIN. Normality of the residuals and homogeneity of variance assumptions were checked using stem-and-leaf box and normal probability plots of the residuals, and Levene's test. Data were not transformed prior to analysis. Treatment means were compared for significance using *t*-tests or Tukey's test at α = 0.05 level.

## Results

### Weather, bulk density, and WFPS

**Figure 1:**
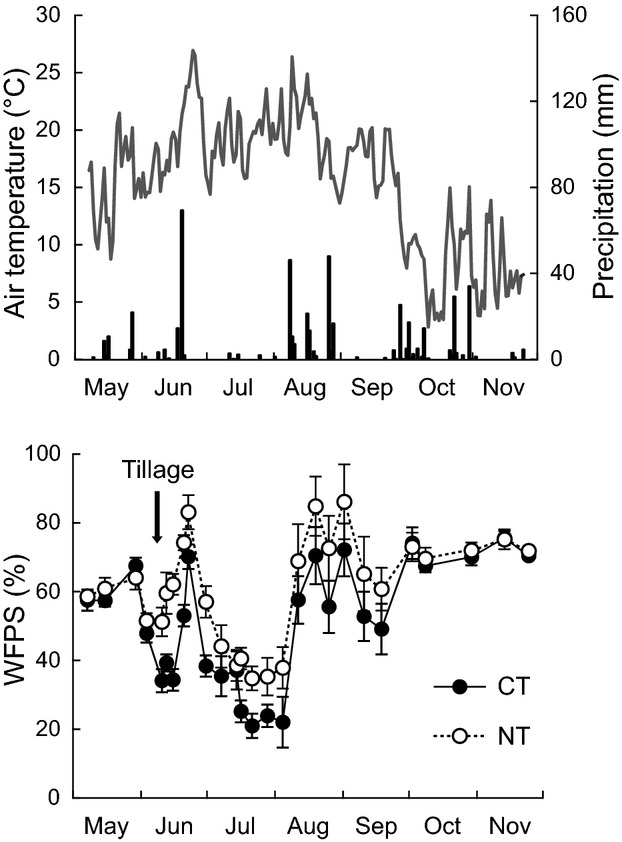
Precipitation and air temperatures (top) and water-filled pore space (WFPS%) to 25 cm depth (bottom) under conventional tillage (CT) and no-till NT soybeans in 2009. Arrow indicates tillage date in the CT treatment.

Air temperature, precipitation, and soil moisture are shown in Fig. 1. Mean daily air temperature was 15.4 °C for the study period of May 3 to November 24, 2009, ranging between 2.8 °C and 26.9 °C. Cumulative precipitation was 443 mm with a drought period from July 1 to August 7, during which time no precipitation >2 mm occurred.

Soil BD (0–25 cm depth) in the CT treatment decreased from 1.51 ± 0.01 to 1.32 ± 0.02 g cm^−3^ after tillage operations on June 8 and gradually increased back to 1.49 ± 0.04 g cm^−3^ by the end of the season. On the other hand, BD in NT and reference treatments stayed stable over the study period at 1.51 ± 0.01 g cm^−3^.

Water-filled pore space (WFPS%) varied between 21.0% and 86.1% with the highest values in June and August and the lowest values in July. No significant differences were found before June 8 (tillage date in CT) between CT and NT treatments. During the 2 months after June 8, average WFPS% in NT was significantly higher than in CT (52% ± 0.04 vs. 36% ± 0.03, respectively, *P* < 0.05). Over June 17–19, 83 mm precipitation occurred and WFPS% under both treatments reached a peak. After a 64 mm precipitation event on August 8, there were no significant differences in soil water content between CT and NT for the remainder of the study.

### Soil N_2_O fluxes

**Figure 2:**
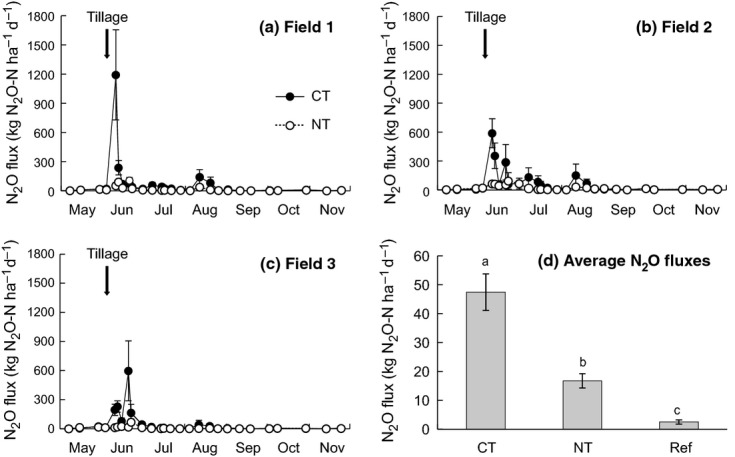
Daily N_2_O fluxes by treatment [conventional tillage (CT) vs. no-till (NT) soybean] in each of three conservation reserve program (CRP) fields (panel a–c) for May 7 to November 24, 2009. Error bars represent standard errors of N_2_O emissions based on *n* = 2 replicate plots. Arrows indicate tillage date. Panel (d) shows average N_2_O fluxes for CT and NT treatments (*n* = 6 replicate plots) and an unconverted CRP reference field (Ref) (*n* = 4 replicate plots). Treatments marked with different letters are significantly different from one another (*P* < 0.01).

**Figure 3:**
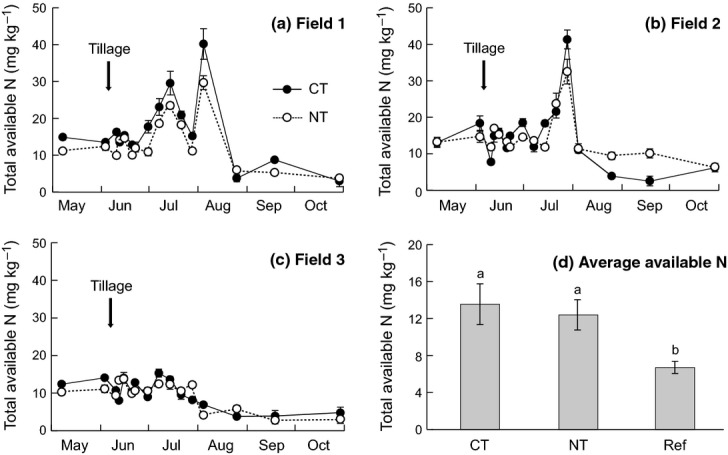
Seasonal dynamics of soil inorganic N pools to 25 cm depth (NH_4_^+^-N plus NO_3_^−^-N) measured in soil cores under conventional tillage (CT) and no-till (NT) soybeans in 2009 (panel a–c). Error bars represent standard errors of total inorganic N based on *n* = 2 replicate plots. Arrows indicate tillage date. Panel (d) shows mean soil inorganic N in CT and NT treatments (*n* = 6 replicate plots) and an unconverted conservation reserve program (CRP) reference field (Ref) (*n *= 4 replicate plots) over the study period. Treatments marked with same letters are not significantly different from one another (*P* < 0.05).

High N_2_O fluxes occurred immediately after CT tillage on June 8, and ranged from 196 to 1192 g N_2_O-N ha^−1^ d^−1^ among the three converted fields. In contrast, on the same date NT fluxes ranged from 10.6 to 63.6 g N_2_O-N ha^−1^ d^−1^ among fields, and in the reference field fluxes ranged from 1.58 to 5.92 g N_2_O-N ha^−1^ d^−1^ (Fig. 2). Tillage-induced fluxes persisted for 30–40 days. Other two relatively large peaks occurred at the converted fields on June 20–22 and August 11 after rainfall events. Subsequently, significant fluxes took place mostly when WFPS in the 0–25 cm depth was greater than 60%. N_2_O emissions from the reference field remained at low levels (<7.21 g N_2_O-N ha^−1^ d^−1^) even after substantial rainfall. After August 25, N_2_O fluxes were low in all fields, coincident with less available soil N (Fig. 3) and lower air temperatures beginning in mid-September (Fig. 1). Soil temperature and WFPS% showed a positive correlation with daily N_2_O fluxes, but the correlation was not significant (*P* > 0.05). Overall, for the May 7 to November 24 period, mean daily N_2_O emissions under CT were 2.8 times those of NT (47.5 ± 6.31 vs. 16.7 ± 2.45 g N_2_O-N ha^−1^ d^−1^; *P* < 0.01) and in both CT and NT treatments rates were substantially higher than in the reference field (2.51 ± 0.73 g N_2_O-N ha^−1^ d^−1^; *P* < 0.01) (Fig. 2d).

### Soil CO_2_ fluxes

**Figure 4:**
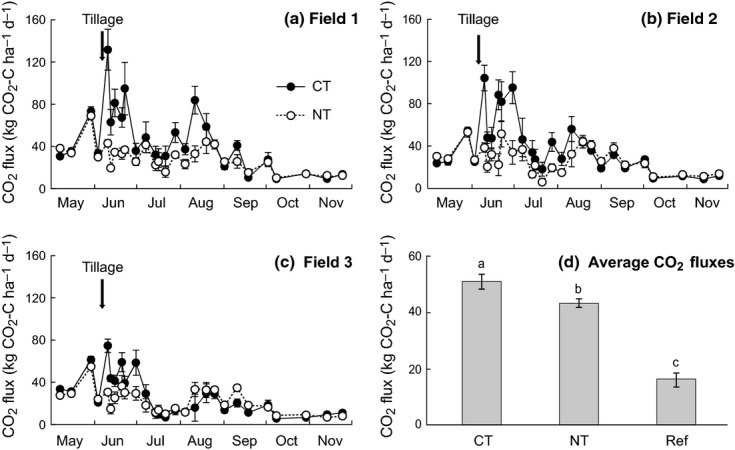
Daily CO_2_ fluxes by treatment [conventional tillage (CT) vs. no-till (NT) soybean] in each of three conservation reserve program (CRP) fields (panel a–c) for May 7 to November 24, 2009. Error bars represent standard errors of CO_2_ emissions based on *n* = 2 replicate plots. Arrows indicate tillage date. Panel (d) shows average CO_2_ fluxes for CT and NT treatments (*n* = 6 replicate plots) and an unconverted CRP reference field (Ref) (*n* = 4 replicate plots). Treatments marked with different letters are significantly different from one another (*P* < 0.05).

Soil CO_2_ fluxes (chamber measurements) showed a seasonal trend in all treatments with high emissions through the growing season and lower emissions after October (Fig. 4), coincident with lower air temperatures (Fig. 1). After herbicide application at the converted fields on May 5, chamber-based CO_2_ fluxes increased sharply and reached a peak on May 29 before tillage started. Immediately following CT tillage on June 8, average CT CO_2_ fluxes on June 8 ranged from 72.2 to 140 kg CO_2_-C ha^−1^ d^−1^, compared to 29.6–43.7 kg CO_2_-C ha^−1^ d^−1^ in the NT treatments (Fig. 4). High fluxes associated with tillage lasted ∼20 days, during which daily fluxes ranged from 0.12 to 168 kg CO_2_-C ha^−1^ d^−1^. Overall, mean CO_2_ fluxes under CT were 1.2 times those of NT (50.7 ± 2.50 vs. 43.0 ± 1.43 g kg CO_2_-C ha^−1^ d^−1^; *P* < 0.05) and were 3.1 times those from the reference (16.3 ± 2.36 kg CO_2_-C ha^−1^ d^−1^; *P* < 0.05) (Fig. 4d). When only comparing the first 30 days after tillage, the CT treatment emitted 2.0 times higher CO_2_ fluxes than did the NT treatment.

### Soil CH_4_ fluxes

**Figure 5:**
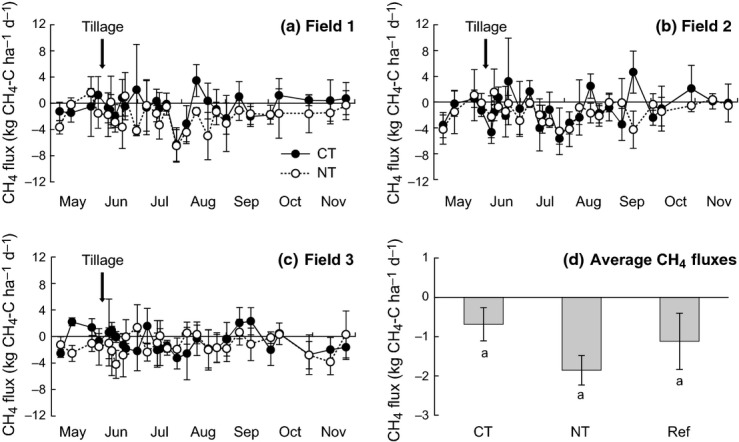
Daily CH_4_ fluxes by treatment (CT vs. no-till (NT) soybean) in each of three conservation reserve program (CRP) fields (panel a–c) for May 7 to November 24, 2009. Error bars represent standard errors of CH_4_ emissions based on *n *= 2 replicate plots. Arrows indicate tillage date. Panel (d) shows average CH_4_ fluxes for CT and NT treatments (*n* = 6 replicate plots) and an unconverted CRP reference field (Ref) (*n* = 4 replicate plots). Treatments marked with different letters are significantly different from one another (*P *< 0.05).

Methane (CH_4_) fluxes oscillated in all fields between net emission and net uptake without a discernable seasonal trend. Mean daily CH_4_ fluxes were low, ranging from −6.4 to 4.5 g CH_4_-C d^−1^ (Fig. 5). Over the entire study period, all treatments exhibited net CH_4_ uptake, but no significant treatment differences were detected. Although mean CH_4_ oxidation rates were 1.7 times higher under NT than under CT (−1.86 ± 0.37 vs. −0.69 ± 0.42 g CH_4_-C ha^−1^ d^−1^, respectively), the difference was not statistically significant (*P* = 0.06). Reference field fluxes also were not significantly different from those in the CT treatment (*P* = 0.32), although uptake in CT soils was only 60% of that in the reference field.

### Grain yield

Soybean grain yield in individual plots ranged from 2.0 to 2.5 Mg ha^−1^. The overall comparison of mean soybean grain yield showed no significant differences between CT (2.4 ± 0.18 Mg ha^−1^) and NT (2.3 ± 0.14 Mg ha^−1^) treatments.

### Global warming impact

As noted earlier, NEE for NT soybean was measured directly as for the reference field.

Because there were no significant yield differences between CT and NT treatments, NEE for CT soybean was calculated as the sum of NEE for NT soybean plus the difference between CT and NT soil CO_2_ fluxes, that is, 10.7 ± 1.37, 6.61 ± 2.02, and 3.66 ± 1.32 Mg CO_2_e ha^−1^ for the three converted fields over the study period.

**Figure 6:**
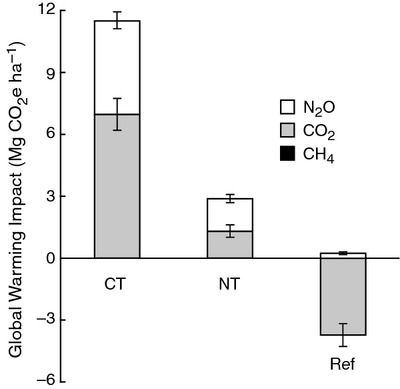
The global warming impact (GWI) of individual greenhouse gases for CT soybean, no-till (NT) soybean and the unconverted conservation reserve program (CRP) reference field (Ref) for May 7 to November 24, 2009. Methane values were negligible and are not visible in graph. For N_2_O and CH_4__,_ error bars represent standard errors based on *n* = 6 replicate plots of CT and NT and *n* = 4 replicate plots for the reference plots; for the net ecosystem exchange (NEE) of CO_2_, error bars represent standard errors based on *n* = 6 replicate plots of CT and *n* = 3 of NT and *n* = 1 for reference fields.

Over our 201 day study period, then, GWIs were 11.5, 2.87, and −3.50 Mg CO_2_e ha^−1^ under CT, NT, and reference treatments, respectively (Fig. 6). Both CT and NT soybean had positive GWIs, with the GWI of CT soybean approximately 2.6 times that of NT soybean (Fig. 6).

### Soil inorganic nitrogen

**Figure 7:**
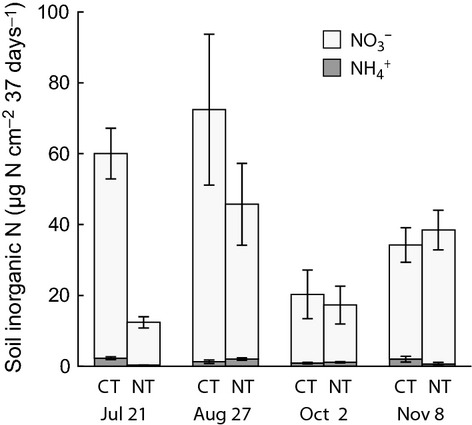
Seasonal dynamics of soil inorganic nitrogen (NH_4_^+^-N + NO_3_^−^-N) measured with cation (NH_4_^+^) and anion (NO_3_^−^) resin strips under CT and NT soybeans. Error bars represent standard errors of NH_4_^+^-N and NO_3_^−^-N based on *n* = 6 replicate plots. Resin strips were buried (0–15 cm depth) for ∼37 days since tillage (June 8, 2009) and then replaced with new pairs three times during the season.

Resin strip results (Fig. 7) showed that tillage greatly increased soil N availability for at least the first month following plowing. Over the 37 day period beginning June 8, strips under CT accumulated 4.8 times more total inorganic nitrogen, mostly as NO_3_^−^, than did strips under NT (60.0 vs. 12.4 μg N cm^−2^). Over the following month, this difference began to diminish (70.4 vs. 45.7 μg N cm^−2^, *P* > 0.05) and there were no discernible differences later in the season. Daily N_2_O fluxes showed a positive relationship with total available N: N_2_O fluxes = 34.8 × EXP (0.36 × available N) (*R*^2^ = 0.19, *P* < 0.01).

In contrast, by the soil-KCl extraction method, soil inorganic N concentrations (0–25 cm depth) (Fig. 3) were significantly higher under CT than NT (16.2 vs. 10.0 mg kg^−1^, *P* < 0.05) in only one field for the first day following tillage and overall results showed no consistent differences (Fig. 3d). Mean total inorganic N concentrations in reference fields were significantly lower than those in the CT and NT treatments (*P* < 0.05) (Fig. 3d). Soil inorganic N concentrations showed a seasonal trend in both treatments with high concentrations through the growing season and lower concentrations after September. Among different fields, soil inorganic N concentrations ranged from a high of 21.6 mg kg^−1^ on June 28 to low values of 3.8–5.1 mg kg^−1^ after September.

## Discussion

The conversion of our CRP grasslands into row crops resulted in a substantial GHG release that differed by tillage practice. The most remarkable difference between CT and NT management during conversion was in N_2_O fluxes. We found immediate and substantial tillage-induced N_2_O emissions under CT that exceeded the CO_2_-equivalent loss of soil C over the 201-day study period. Total N_2_O emissions under converted CT soybean were 2.1-fold higher than under converted NT soybean and 18.8-fold higher than under unconverted smooth brome grass (reference field). The magnitude of CT N_2_O emissions exceeded that of fertilizer-induced N_2_O fluxes in the same area (Robertson *et al*., 2000; Hoben *et al*., 2011). Even with NT practices, however, CRP conversion still caused large N_2_O emissions, with fluxes under NT 5.3 times higher than under unconverted reference.

Soil CO_2_ emissions under CT were also significantly higher than those under NT and reference treatments. Cumulative NEE of CO_2_ under CT were 2.2-fold higher than those under NT over the study period. The converted fields under both CT and CT were carbon sources under both CT and NT, whereas the unconverted reference treatment was a net carbon sink. All treatments were a small sink for atmospheric CH_4_. However, changes in CH_4_ oxidation rates did not contribute significantly to the GWI of conversion compared with N_2_O and CO_2_. Overall, N_2_O accounted for 39.3% of the net GWI of conversion under CT and 55.0% under NT with the remainder contributed by CO_2_ (60.7% and 45.0%, respectively), excluding the CO_2_ costs of herbicide and fuel, which were negligible (Gelfand *et al*., 2011).

### N_2_O emissions

Nitrous oxide (N_2_O) fluxes increased 18- to 55-fold immediately on the first day after tillage operations in all CT treatments. Over the study period, mean daily CT N_2_O emissions (47.5 ± 6.3 g N_2_O-N ha^−1^ d^−1^) were relatively higher than those reported for fertilized annual crops at a nearby site (3.35 ± 0.30 g N_2_O-N ha^−1^ d^−1^) (Robertson *et al*., 2000) and for heavily fertilized crops elsewhere in Michigan (25.8 g N_2_O-N ha^−1^ d^−1^from corn fertilized at 225 kg N ha^−1^) (Hoben *et al*., 2011). Similar substantial amounts of N_2_O emissions following tillage have been reported for other studies where unmanaged vegetation has been converted to cropland. For example, Grandy & Robertson (2006a) reported a 3.1 to 7.7 fold increase in N_2_O emissions after plowing long-term undisturbed grassland over a 3 year period. Nikièma *et al*. (2012) reported high N_2_O fluxes of 57.2 and 41.8 g N_2_O-N ha^−1^ d^−1^ after converting heavily manured pastureland (200 kg N ha^−1^ yr^−1^) to poplar and willow production, respectively. Possible reasons for high N_2_O emissions could be increased production of available N and C after SOM mineralization (Grandy & Robertson, 2006a) and increased substrate supply to nitrification and denitrification after the incorporation of residues into the soil (Piva *et al*., 2012). In contrast, daily N_2_O fluxes under NT also continuously increased from 1.93 ± 0.75 to 66.7 ± 16.0 g N_2_O-N ha^−1^ d^−1^ for the first 45 days after herbicide application, but overall rates were approximately one third of those from under CT. Available C and N from decomposed dead grass and roots are likely reasons.

In the unfertilized fields studied here, available N could be one of the most important driving factors for N_2_O emissions. Accelerated N mineralization from SOM and incorporated residue after tillage can increase available N and thus enhance nitrification and denitrification. Resin strip measurements indicate that for the 37 day period after CT tillage, soil NO_3_^−^-N and NH_4_^+^-N concentrations under CT (57.7 ± 7.16 and 2.30 ± 0.41 μg N cm^−2^) were substantially higher than those under NT (12.1 ± 1.59 and 0.31 ± 0.08 μg N cm^−2^), respectively. Daily N_2_O fluxes were strongly correlated with total available N from resin strip measurements (N_2_O fluxes = 34.8 × EXP (0.36 × available N), *R*^2^ = 0.19, *P* < 0.01). However, NO_3_^−^-N and NH_4_^+^-N concentrations in soil cores showed no consistent differences between CT and NT. This is likely because soil-KCl extractions measure only the soil available N pool size. This pool can be rapidly utilized by microbes and plants or leached out of the soil so that it cannot be detected accurately, especially when the N pool size is small. In contrast, ion exchange strips measure both the soil available N pool and the flux of N ions through the mineral pool (Bowatte *et al*., 2008). In this study, the resin strips provided the more interpretable results.

Soil N_2_O fluxes were also likely affected by available soil carbon (Dalal *et al*., 2003; Wang *et al*., 2011). Firstly, killed and incorporated brome grass, in conjunction with dead roots, provided heterotrophic denitrifiers with more available carbon and as well will have increased O_2_ demand. CO_2_ as an end product of decomposition indicated the extent to which dead brome grass was decomposed. Especially during the period between herbicide application (May 5) and tillage operations in the CT treatment (June 8), soil CO_2_ emissions were 5.7 times, those of emissions from the unconverted reference field, indicating that more decomposition took place in the herbicide applied fields than in the reference field. In addition, the old CRP land had accumulated relatively high amounts of SOC, which has a potential to provide more available carbon for N_2_O production due to SOC decomposition after tillage. Compared to SOC at nearby LTER experimental sites (Syswerda *et al*., 2011), SOC concentrations in our studied fields (21.3 ± 0.8 g C kg^−1^ soil) prior to the conversion were comparatively higher than annual crops under CT (10. 4 ± 3.4 g C kg^−1^ soil) and NT (11.5 ± 0.4 g C kg^−1^ soil) and close to deciduous forest levels (24.0 ± 3.4 g C kg^−1^) for 0–20 cm depth. In addition, enhanced SOM decomposition will consume oxygen and create localized anaerobic conditions favoring denitrification (Wang *et al*., 2011).

Soil N_2_O fluxes are also affected by soil water content. Two relatively larger N_2_O peaks occurred after rainfall in this study when WFPS% was >60%. The possible reason is that rainfall events create anaerobic conditions, which can stimulate N_2_O emissions from denitrification. This finding has been reported by many studies (e.g., Elder & Lal, 2008; Wang *et al*., 2011). However, in this study overall N_2_O fluxes showed no significant correlation with soil moisture (*P* > 0.05). Wet soil conditions did not necessarily give rise to high N_2_O emission. For example, soil N_2_O fluxes in the reference field remained low and stable through the whole study period even after considerable rainfall. In addition, we observed low emissions of N_2_O at all fields after September even when WFPS% was larger than 60% following rainfall. For both cases, this indicates that N_2_O production was likely restricted by other more limiting factors such as available N or low temperature.

The comparison between NT and CT N_2_O fluxes has been widely studied and it is still difficult to generalize. Six *et al*. (2004) analyzed 44 comparisons of N_2_O emissions under CT and NT globally and found higher N_2_O emissions in the first 10 years of NT than CT and thereafter similar or lower N_2_O emissions under NT. They argued that increased soil water content under NT promoted denitrification and thus enhanced N_2_O production in the first 10 years. A more recent study using a meta-analysis of 239 direct comparisons between CT and NT/reduced tillage (Van Kessel *et al*., 2013) found no N_2_O emission differences. However, in this study, CRP land with its long-term no-till history and high SOC may provide a special case. Our results suggest that adopting NT practices can significantly reduce N_2_O emissions compared to CT, but NT management cannot eliminate the cost of N_2_O emissions during CRP conversion.

### CO_2_ emissions

Soil CO_2_ emissions under both CT and NT soybeans were significantly higher than those in unconverted reference fields (*P* < 0.05). Two possible reasons are (i) decomposition of dead grass and roots in the soil; and (ii) accelerated SOM decomposition after tillage. In addition, soil CO_2_ emissions in CT soybean were higher than emissions in NT soybean (*P* < 0.05). Similar results have been reported in many studies (e.g., Grandy & Robertson, 2006a; Chatskikh & Olesen, 2007; Alluvione *et al*., 2009). Tillage enhanced SOC decomposition and thus increased CO_2_ release to the atmosphere.

Soil CO_2_ fluxes can be governed by soil temperature, moisture, and other factors. Multiple Linear regressions of soil CO_2_ fluxes with soil temperature and WFPS% showed no significant correlation between CO_2_ fluxes and WFPS%, although WFPS% might have affected CO_2_ emission at some specific times during the drought period in July with its relatively low emissions. On the other hand, a positive relationship was found between soil CO_2_ fluxes and soil temperature: soil CO_2_ fluxes = 11.5 × EXP (0.07 × soil temperature), *R*^2^ = 0.21, *P* < 0.01). Exponentially increased soil CO_2_ fluxes with rising temperature have been reported by many studies (e.g., Lloyd & Taylor, 1994; Reichstein & Beer, 2008; Almaraz *et al*., 2009).

The NEE of CO_2_ fluxes for CT soybeans was more than twice that for NT soybeans, and the converted fields under both CT and NT were net sources for CO_2_. This is because carbon released from the decomposition of grass residue and SOC exceeded the carbon uptake from photosynthesis in converted fields. On the contrary, the unconverted reference field was a net sink for atmospheric CO_2_.

### CH_4_ emissions

The range of daily CH_4_ fluxes (−6.4 to 4.5 g CH_4_-C ha^−1^ d^−1^) we observed were similar to CH_4_ fluxes of −1.80 ± 0.06 g CH_4_-C ha^−1^ d^−1^ for cropland in Michigan (Robertson *et al*., 2000). All fields were net sinks for CH_4_, although some other studies found cropland under CT could be a small net source (Alluvione *et al*., 2009; Ussiri *et al*., 2009). Fluxes in CO_2_ equivalents were negligible compared with CO_2_ and N_2_O fluxes, which had generally been reported for other upland cropping systems (Robertson *et al*., 2000; Wang *et al*., 2011).

No statistically significant differences in CH_4_ oxidation rates were found among any treatments, although oxidation rates in CT were 62.9% and 38.8% lower than those in the NT and reference treatments, respectively. Similar results of no differences between CT and NT systems have been reported in some studies for sites nearby (Robertson *et al*., 2000; Suwanwaree & Robertson, 2005). However, other studies reported higher oxidation rates in NT than CT or uptake in NT but net emissions in CT (Ussiri *et al*., 2009). They attributed this to undisturbed soil structure and greater gas diffusion under NT. Another possible reason was that increased mineralization after tillage enhanced NH_4_^+^ production, and NH_4_^+^ could competitively inhibited CH_4_ oxidation. In addition, we found no significant difference in CH_4_ oxidation before and after conversion of CRP land, although some studies have found that the CH_4_ oxidation rates of a grassland were reduced by 75% after only 8 months of conversion into CT cropland (Ball *et al*., 1999) or higher CH_4_ oxidation rates in midsuccessional grassland than cropland (Robertson *et al*., 2000). It seems likely that CH_4_ oxidation rates had not increased under 20 years of CRP brome grass sufficiently to be significantly re-suppressed by cropping.

Methane (CH_4_) oxidation rates can also be regulated by soil water content and soil temperature. CH_4_ oxidation rates were found negatively correlated with soil water content in some studies, probably due to limited CH_4_ diffusion in the wet soil (Del Grosso *et al*., 2000; Khalil & Baggs, 2005). However, CH_4_ oxidation may be inhibited in dry soils (Khalil & Baggs, 2005). In this study, no apparent seasonal CH_4_ flux patterns were observed. We found CH_4_ fluxes were not significantly related with either WFPS% or soil temperature in any treatments, although other studies have shown CH_4_ flux from NT to be negatively correlated with soil temperature (Ussiri *et al*., 2009).

### Global warming impact

Over the study period (201 days), the GWI of converted soybean fields was 11.5 and 2.87 Mg CO_2_e ha^−1^ for CT and NT operations, respectively, whereas the GWI of the unconverted CRP reference field was −3.5 Mg CO_2_e ha^−1^(Fig. 6). The positive GWI of the converted fields indicates net GHG emissions to the atmosphere, while the negative GWI in the reference field indicates on-going GHG mitigation. The possibility that increased N_2_O emissions might offset the enhanced soil carbon sequestration in NT systems has been a concern for adopting NT practices (Six *et al*., 2002; Li *et al*., 2005), but this was not the case in this study. NT played an important role in reducing GWI compared to CT, by significantly decreasing N_2_O emissions and reducing SOC loss.

The CT system exhibited a net positive GWI of 11.5 Mg CO_2_e ha^−1^. In this system, about 39.3% of the GWI was contributed by N_2_O production (4.52 Mg CO_2_e ha^−1^) even in the absence of synthetic N fertilizer additions. SOC loss as indicated by net CO_2_ emissions contributed the remainder (60.7% or 6.98 Mg CO_2_e ha^−1^). For the NT system, net GWI was 2.87 Mg CO_2_e ha^−1^, about 55.0% of which was contributed by N_2_O production (1.57 Mg CO_2_e ha^−1^) with the remaining 45% from CO_2_ emissions (1.30 Mg CO_2_e ha^−1^). The contribution of CH_4_ oxidation was negligible (<0.1%) under both CT and NT systems.

In contrast to converted fields, the unconverted reference fields showed a net mitigation potential of −3.50 Mg CO_2_e ha^−1^ due to very low rates of N_2_O production and a net uptake of CO_2_.

The net mitigation potential for the unconverted reference fields indicates that the conversion of CRP land not only increases the emissions of GHGs but also causes the loss of the CRP land's net GHG mitigation ability: 3.5 Mg CO_2_e ha^−1^ mitigation would have happened had no conversion occurred. This foregone mitigation capacity must be added to the post conversion GHG fluxes to provide a total net GWI (Gelfand *et al*., 2011). This yields a total initial cost of 6.4 Mg CO_2_e ha^−1^ for NT and 15.0 Mg CO_2_e ha^−1^ for CT soybean. Thus, NT can reduce GHG costs by ∼60% as compared to CT.

Robertson *et al*. (2000) calculated for a nearby site under the same soil series that NT practices sequestered 30 g C m^−2^ yr^−1^. Based on this rate, CRP conversion by CT rather than NT cost ∼8 years of NT carbon sequestration with a single tillage event.

Over time, this additional cost will change depending on future management. If planted with perennial biofuel crops (no tillage and no N fertilization), the plowed soils will stop losing and begin re-accumulating soil carbon and N_2_O fluxes will likely be low. In contrast, if planted with annual grain crops that are plowed and fertilized every year, soil carbon will continue to be lost until the soil equilibrates (to ∼10.4 g C kg^−1^ soil from annual crops under CT at the nearby KBS LTER site). N_2_O production differences due to CT and NT will likely diminish (Van Kessel *et al*., 2013) but N_2_O fluxes will continue to be high due to N fertilization (Hoben *et al*., 2011).
